# Rediscovering the human thymus through cutting-edge technologies

**DOI:** 10.1084/jem.20230892

**Published:** 2024-08-21

**Authors:** Francesca Pala, Luigi D. Notarangelo, Marita Bosticardo

**Affiliations:** 1 Immune Deficiency Genetics Section, Laboratory of Clinical Immunology and Microbiology, National Institute of Allergy and Infectious Diseases, National Institutes of Health, Bethesda, MD, USA

## Abstract

Recent technological advances have transformed our understanding of the human thymus. Innovations such as high-resolution imaging, single-cell omics, and organoid cultures, including thymic epithelial cell (TEC) differentiation and culture, and improvements in biomaterials, have further elucidated the thymus architecture, cellular dynamics, and molecular mechanisms underlying T cell development, and have unraveled previously unrecognized levels of stromal cell heterogeneity. These advancements offer unprecedented insights into thymic biology and hold promise for the development of novel therapeutic strategies for immune-related disorders.

## Teaser

This review highlights how novel cutting-edge technologies, including high-resolution imaging, single-cell omics, and organoid cultures, have improved our understanding of human thymus biology, in terms of developmental trajectories, cellular heterogeneity, and spatial organization.

## Introduction

The human thymus has long captivated the interest of researchers due to its crucial role in T cell maturation and immune tolerance. Yet, studying the thymus has posed challenges owing to its deep anatomical location and dynamic cellular interactions. Before the groundbreaking study of Jacques Miller in 1961 (Miller, 1961), the thymus was considered to be a vestigial organ with no known function, which had become redundant during the course of evolution and was simply serving as a graveyard for dying lymphocytes. Since then, our knowledge of the structure, development, and function of the thymus has greatly improved (Dinges et al., 2024; Pala et al., 2022). Briefly, early T cell progenitors (ETPs) originating from bone marrow precursors migrate to the thymus, where they undertake a series of developmental steps. In the outer cortex, thymocytes proliferate rapidly and undergo positive selection. This process involves successful rearrangement of T cell receptor (TCR) genes and the recognition of self-antigens presented by thymic epithelial cells (TECs) in complex with major histocompatibility complex (MHC) molecules. Thymocytes that have successfully initiated the process of TCR rearrangement and passed positive selection can then progress to the next stage of development in the inner medulla, where they undergo negative selection (Ashby and Hogquist, 2024). During this process, thymocytes encounter self-antigens presented by medullary TECs (mTECs) and dendritic cells (DCs). Thymocytes with TCRs that bind strongly to self-antigens undergo negative selection through apoptosis, thus preventing export of self-reactive T cells. Thymocytes expressing TCRs with intermediate affinity for self-antigens differentiate into regulatory T cells (Treg) crucial for immune tolerance. Thymocytes that successfully pass both positive and negative selection differentiate into mature T cells that exit the thymus and enter circulation to participate in immune responses throughout the body.

While these fundamental concepts have been known for decades, recent technological advancements have shed new light on the thymus’ intricate architecture and functional dynamics (Bornstein et al., 2018; Givony et al., 2023; Michelson et al., 2022, 2023; Miller et al., 2018). Historically, studies on thymus biology have used histology, immunohistochemistry, and flow cytometry in human specimens, or taken advantage of results obtained using mouse models, where the thymus is easier to isolate and study in its entirety. Histology and immunohistochemistry have allowed to elucidate the macroscopic structure of the human thymus, with the distinction of cortex, medulla, capsular and subcapsular areas, vasculature, and Hassall’s corpuscles (HCs), in addition to localizing different thymocyte developmental stages across these areas. However, the limited number of known markers and the low resolution did not allow for fully uncovering the heterogeneity of the different compartments, both stromal and hematopoietic. Flow cytometry introduced the possibility of looking at multiple markers at the same time at the single-cell level, and it has been particularly useful in the study of thymocytes. However, a major limitation of flow cytometry has consisted of the lack of well-defined markers to identify all the cell subsets composing the human thymus, in particular in the stromal compartment. Mouse models have been an invaluable instrument to characterize the developmental trajectories of thymocytes, as well as to study genes and molecular mechanisms involved in T cell and stromal cell development and function (Miller, 2011). Nevertheless, genetic differences, as well as dissimilar lifespan and environmental cues at play in mice and humans, have often impeded a direct translation of these findings to human biology. For these reasons, the introduction of novel imaging and transcriptomics techniques allowing single-cell and spatial resolution represents a huge step forward in the comprehension of human thymus biology (main techniques covered in this review are summarized in Table 1). In particular, high-resolution imaging techniques and 3D reconstructions have provided unprecedented insights into the thymic microenvironment, enabling the visualization of cellular interactions in real-time. Single-cell omics technologies, including single-cell RNA sequencing (scRNA-seq) and multiparametric flow cytometry, have unveiled the heterogeneity of thymic cell populations and their transcriptional profiles, thus further elucidating the molecular mechanisms underlying T cell development and selection. Although in this review we will focus mainly on conventional and unconventional T cells and stromal cells, several other lymphoid and myeloid cell types have also been identified and characterized, including eosinophils, B cells, and plasma cells (Albinsson et al., 2023; Castañeda et al., 2021; Nuñez et al., 2016). Furthermore, the emergence of organoid culture systems has facilitated the recapitulation ex vivo of T cell and thymic stromal cell development and enabled the monitoring of thymic function in human primary cells. Harnessing the power of these cutting-edge technologies offers promising avenues for unraveling the complexities of thymic biology and holds implications for the development of novel therapeutic strategies targeting immune-related disorders. This review underscores the transformative impact of recent technological advances in improving our understanding of the human thymus and highlights the potential avenues for future research in this field.

**Table 1.** Single-cell and spatial omics methods

Source material	Method	Technologies^a^	Data type	Advantages	Limitations
Dissociated tissue	Plate-based sequencing	Smart-Seq2	mRNA	Generate full-length transcripts, allow detection of genes that are not highly expressed and other rare transcripts	Limited by plate size and the quantity of cells available for analysis
	Droplet-based microfluidics sequencing	10X Genomics 3′ scRNA-seq	mRNA	Higher throughput (thousands of cells), easy to multiplex	Only from the 5′ or 3′ end of the transcript, omitting the detection of allele-specific expression and other isoforms
		10X Genomics 5′ scRNA-seq + scTCR-seq	mRNA, T cell receptor clonotype		
		CITE-seq	mRNA, protein		
		scATAC-seq (10X Genomics Multiome)	mRNA, chromatin accessibility		
Tissue section	Microarray-based sequencing	10X Genomics Visium	Spatial, mRNA (55 μm)	Unbiased, probes include 18,000 unique genes	Low capture efficiency, low resolution
	Imaging-based	Phenocycler (CODEX)	Spatial, protein (subcellular resolution, DNA oligo-tagged antibodies)	High resolution (single cell and subcelluar), retains information on physical interactions within and between cells, quantitatively characterizes protein expression	Readout limited to targeted markers (14 for RareCyte, 50–60 for CODEX and IBEX), iterations needed to collect multiple markers, antibody validation needed
		IBEX	Spatial, protein (subcellular resolution, flurochrome-tagged antibodies)		
		RareCyte	Spatial, protein (subcellular resolution, flurochrome-tagged antibodies)		

a Non exhaustive list, limited to technologies used in the cited literature.

## Single-cell omics: Down to the nitty-gritty details

### Hematopoietic cell compartment

#### Thymocyte precursors

**Figure 1.**
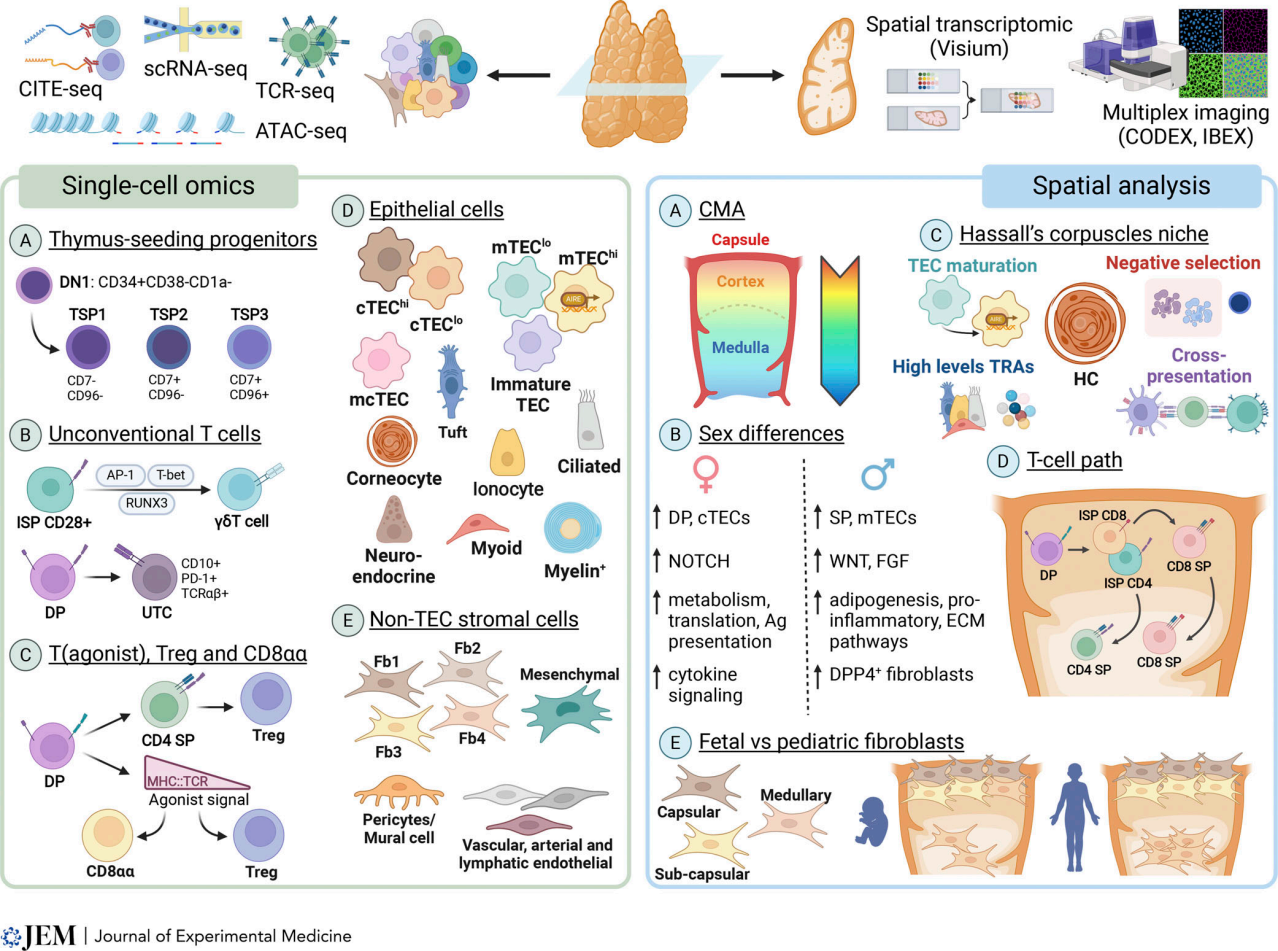
**Main advancements on human thymus from single-cell omics and spatial analysis.** The left panel highlights significant findings obtained through single-cell omics studies performed on dissociated thymic tissue, including scRNA-seq, CITE-seq, TCR-seq, and ATAC-seq. **(A)** Thymus seeding progenitors based on Cordes et al. (2022). **(B)** Unconventional T cells based on Billiet et al. (2023); Roels et al. (2020). **(C)** T(agonist), Treg, and CD8αα based on Heimli et al. (2023). **(D)** TEC populations based on Bautista et al. (2021); Campinoti et al. (2020); Park et al. (2020). **(E)** Non-TEC stromal cells based on Heimli et al. (2023); Park et al. (2020); Ragazzini et al. (2023). The right panel shows novel insight into thymus biology deriving from studies using spatial transcriptomics and multiplex imaging. **(A)** CMA based on Yayon et al. (Yayon et al., 2023, *Preprint*). **(B)** Sex differences based on Stankiewicz et al. (Stankiewicz et al., 2023, *Preprint*). **(C)** HC’s niche based on Stankiewicz et al. (Stankiewicz et al., 2023, *Preprint*) and Yayon et al. (Yayon et al., 2023, *Preprint*). **(D)** T cell developmental pathway based on Yayon et al. (Yayon et al., 2023, *Preprint*). **(E)** Differences in fibroblast localization in fetal and pediatric thymus based on Yayon et al. (Yayon et al., 2023, *Preprint*).

The quest to identify human thymic precursors and ETPs has remained an open issue for many years. Thymus-seeding progenitors have been hard to identify in humans due to the relative scarcity of these cells and the lack of known markers to isolate and characterize them. To this end, Lavaert and colleagues (Lavaert et al., 2020) employed a methodology combining scRNA-seq and cellular indexing of transcriptomes and epitopes by sequencing (CITE-seq), which allowed them to correlate the transcriptome with cell surface marker expression. Focusing on the immature postnatal thymocytes, they isolated CD34^+^ thymocytes from infants. By integrating this dataset with datasets from CD34^+^ cells isolated from bone marrow and peripheral blood, they identified two potential thymus seeding progenitors (TSPs) distinguished by varying expression levels of *CD7*, *CD10*, and the homing receptors *CCR7*, *CCR9*, and *ITGB7*. TSP1 (CD34^+^CD10^+^CD7^−^CCR7^+^CCR9^+^ITGB7^−^) represent the canonical T cell precursors that differentiate into ETPs when they encounter Notch activating signals within the thymic microenvironment, while TSP2 (CD34^+^CD10^+^CD7^+^CCR7^+^CCR9^−^ITGB7^+^) resemble Notch-primed TSPs, as they express the Notch target genes *CD7* and *CD3E* prior to thymic entry (Lavaert et al., 2020). Similarly, Le and colleagues employed scRNA-seq to examine both the infrequent CD34^+^ progenitors and the comparatively more developed CD34^−^ cells within the human postnatal thymus. Their findings shed light on a process of multilineage priming preceding gradual commitment to the T cell lineage during the early phases of thymopoiesis (Le et al., 2020). In a later effort to further define the progenitor potential of CD34^+^ cells in the human thymus, Cordes and colleagues conducted extensive immune profiling of cells at early stages of prelineage commitment, utilizing a combination of single-cell techniques and functional assays (Cordes et al., 2022). Their analysis revealed three progenitor populations that seed the thymus, identified as TSP1, TSP2, and TSP3, that correspond to bone marrow hematopoietic stem cell–like, multipotent progenitor–like, and common lymphoid progenitor–like TSPs, respectively, and whose potential to give rise to multiple lineages gradually decreased from TSP1 to TSP3. Upon isolating these TSPs based on surface markers (TSP1: CD2^−^CD96^−^CD7^−^, TSP2: CD2^−^CD96^−^CD7^+^, and TSP3: CD2^+^CD96^+^CD7^+^) (Fig. 1 A, left), Cordes et al. (2022) demonstrated that they have distinct T cell proliferation and differentiation kinetics, with the TSP1 being a quiescent, nonproliferating population, and the TSP3 cells primed to develop much more rapidly into T cells. These three studies also identified a TSP population that can generate plasmacytoid DCs (pDCs) in the thymus. In Lavaert’s study, the development of pDCs was observed to occur via an intermediate stage characterized by high expression of IRF8 within the granulocyte–macrophage progenitor precursor. This intermediate stage is likely to emerge within the thymus from the TSP2 subset, influenced by both Notch and type I interferon (IFN-I) signaling pathways (Lavaert et al., 2020). Recently, the same group has demonstrated that IRF8-dependent DC-biased precursors express membrane-bound TNF, which activates TNF receptor (TNFR) 2 rather than TNFR1, promoting the differentiation of thymus seeding hematopoietic progenitors into T lineage specified precursors (Liang et al., 2023). Functional studies mimicking TNFR2 signaling enhanced the generation of human T cell precursors, highlighting the role of DCs as hematopoietic stromal cells that support early human T cell development alongside their roles in thymocyte selection and maturation (Liang et al., 2023).

#### Thymocyte development

T cell development has been studied extensively in mice, but less so in humans. In a collaborative effort to build a Human Thymus Atlas, Park and colleagues (Park et al., 2020) performed scRNA-seq in human thymus samples obtained during fetal life, childhood, and adult life. By applying enrichment strategies to increase the coverage of under-represented cell populations, they were able to identify 50 distinct cell subpopulations in the human thymus. Within the hematopoietic compartment, differentiating T cells were well represented in the dataset, including CD4^−^ CD8^−^ double negative (DN), CD4^+^ CD8^+^ double positive (DP), CD4^+^ single positive (CD4^+^ SP), CD8^+^ SP, FOXP3^+^ Treg, CD8αα, and γδ T cells, as well as other immune cells including B cells, natural killer cells, innate lymphoid cells (ILCs), macrophages, monocytes, and DCs. The latter were further classified into myeloid/conventional DC1 and DC2 and pDC. The additional layer of information provided by scTCR-seq allowed for reconstructing the T cell differentiation process and to define lineage progression by following the sequence of rearrangements in the TCR loci. By overlaying hallmark genes of T cell differentiation (*CD4*/*CD8A*/*CD8B* genes, cell cycle [*CDK1*], and recombination [*RAG1*] genes) and fully recombined TCRα/TCRβ transcripts to the predicted pseudo-time trajectory, Park et al. (2020) identified novel transcriptional signatures associated with different stages of T cell development. They also established novel developmental markers, such as *ST18*, for early DN, *AQP3* for DP, and *TOX2* for DP-to-SP transition. A major contribution of this effort was the identification of clusters of transcription factors that drive T cell lineage commitment and differentiation, although the authors did not address whether changes in the expression of transcription factors would directly correlate with their activity.

#### Agonist T cell development

The Human Thymus Atlas project (Park et al., 2020) delineated the framework on which more groups (Chopp et al., 2020; Morgana et al., 2022; Heimli et al., 2023) worked to better define developmental trajectories and the signals required for CD4 or CD8 SP T cell maturation, as well as the role of agonist signals in the selection and induction of Tregs. Heimli and colleagues identified the αβT(entry) stage as a critical juncture where cells exhibit a transcriptional signature indicative of T cell activation, marking a pivotal point in lineage specification. This stage appears to serve as a nexus from which two prominent branches diverge, leading either to a CD8αα fate or to a T(agonist) trajectory, characterized by gradual shifts in transcriptional profiles reflecting TCR signaling strength and lineage-specific marker expression. These findings are in line with prior research by Chopp and colleagues, emphasizing the significance of developmental time points preceding CD4^+^ or CD8^+^ lineage commitment in the context of agonist selection (Chopp et al., 2020). The expression of *RUNX3* and *THPOK* in human (but not mouse) CD4^+^ thymocytes suggests evolutionary alterations in the nuclear machinery governing lineage commitment (Cleveland and Huseby, 2020). Contrary to previous assumptions, Chopp and colleagues showed that the CD4^+^ lineage gene signature emerges already at the DP-2 stage in human thymocytes, preceding downregulation of *CD8B* expression. While the CD8^+^ lineage gene signature begins later in mice, coinciding with *Cd8* gene re-expression, this distinction is not observed in human CD8^+^ thymocytes. These findings suggest that in humans, CD4^+^ lineage commitment may initiate during positive selection and is potentially influenced by heightened TCR signal strength of MHC-II–restricted thymocytes, whereas CD8^+^ lineage commitment occurs later in development (Chopp et al., 2020).

Contrasting data exist in the literature regarding Treg precursor populations in the human thymus. Morgana and colleagues proposed a model of continuous development from T(agonist)-like populations to Treg-like states (Morgana et al., 2022), while Park and colleagues suggest distinct developmental pathways akin to murine models (Park et al., 2020). These discrepancies underscore the complexity of thymic T cell ontogeny and highlight the need for further investigations. Furthermore, the presence of CD4^+^CD8^+^ DP cells within the T(agonist) branch raises intriguing questions regarding their potential role in Treg induction. While murine models primarily implicate mature CD4^+^ SP thymocytes in Treg generation, evidence of *FOXP3* upregulation in human DP cells suggests alternative pathways worthy of exploration. Heimli and colleagues provide valuable insights into the development of Treg cells (Heimli et al., 2023). Although in their study *FOXP3* expression was detected among late T(agonist) cells, with further increase in expression in differentiating Treg (Treg[diff]) and in mature Treg, pseudotime analysis with cell cycle regression indicated that both Treg(diff) and Treg arise after CD4^+^ SP lineage divergence. According to these data, T(agonist) and Treg(diff) represent distinct progenitor types to mature Treg cells, with T(agonist) to Treg differentiation being induced by high TCR signaling prior to SP lineage commitment, whereas Treg(diff) to Treg differentiation can be induced by a variable range of TCR signaling, but only after commitment to the CD4^+^ SP lineage (Fig. 1 C, left).

Together, all these studies elucidating distinct lineage trajectories and their potential implications for thymocyte differentiation pave the way for future investigations aimed at unraveling the intricacies of immune regulation and tolerance induction within the human thymus.

#### Unconventional T cells

Access to large human thymus single-cell datasets allowed to better define development trajectories of unconventional T cells. The combination of scRNA-seq with TCR-seq, both for the αβ and γδ rearrangements, shed some light on the point of divergence for the development of γδT cells. A combination of RNA-seq, Assay for Transposase-Accessible Chromatin (ATAC)-sequencing, and Chromatin immunoprecipitation (ChIP) combined with sequencing library preparation by Tn5 transposase (ChIPmentation) identified the CD34^+^CD4^+^ subset as the primary precursor population for the γδ lineage, implying that most γδT cells diverge from β-selected immature SP CD28^+^ αβ-lineage precursors during this phase of human T cell development (Roels et al., 2020) (Fig. 1 B, left). Developing γδT cell had also a peculiar gene signature, including *RUNX3*, *TBX21*, and *ZBTB16*, as well as *SOX*, *EGR*, and IL-7R signaling components *STAT5A*, *STAT5B*, and *JAK3*, and a distinctive chromatin landscape with increased accessibility in regions mostly enriched for AP-1–, T-bet–, and RUNX3-binding sites. In a recent study, Sanchez Sanchez and colleagues (Sanchez Sanchez et al., 2022) found that mature fetal γδ thymocytes, including both Vγ9Vδ2 and non-Vγ9Vδ2 subsets, exhibit distinct effector fates (type 1, type 3, or type 2-like) in a gestation age–dependent manner. These effector modules display different CDR3 sequences and developmental trajectories. In contrast, the pediatric thymus generates a small effector subset predominantly utilizing Vγ9Vδ2 TCR and exhibiting a mixed type 1/type 3 effector profile. Perriman and colleagues who investigated the developmental pathway of Vγ9Vδ2 T cells in the postnatal thymus, identified three distinct stages of development characterized by changes in functional potential and expression of key molecules (Perriman et al., 2023). Crucial transcription factors like *TBX21* and *EOMES*, which are required for IFN-γ and TNFα expression, are upregulated during maturation, leading to increased cytokine expression upon stimulation. Additionally, mature thymic Vγ9Vδ2 T cells express cytolytic molecules, such as perforin, granzyme A, and granzyme K, suggesting their potential role in immune responses.

Billiet, De Cock, and colleagues identified a polyclonal unconventional T cell (UTC) population characterized by the expression of CD10, PD-1, and TCRαβ (Billiet et al., 2023) (Fig. 1 B, left). This population deviates from the conventional SP T cell lineages during the early DP stage. Both in the thymus and cord blood, the progeny of these UTCs exhibit similar transcriptomic profiles, marked by the expression of *ZNF683* and *IKZF2* transcription factors, and a diverse TCR repertoire with features suggestive of autoreactivity, including a bias toward early TCRα chain rearrangements. scRNA-seq confirmed a shared developmental trajectory between thymic and blood UTCs, offering a clear delineation of this unconventional lineage in the blood. Additionally, within this heterogeneous lineage, effector-like clusters have been identified alongside recent thymic emigrants expressing *MME*, with distinct characteristics such as the expression of Helios and KIR, as well as reduced CD8β expression, which is related to activation by self-antigens in the thymus.

### Stromal cell compartment

Historically, studies on human thymus have largely focused on the more abundant and easier-to-isolate hematopoietic cells, with efforts on stromal cells being mostly limited to histological characterization. TECs, fibroblasts, and endothelial and mesenchymal cells are the main cellular components of the thymic stroma. More recently, the advent of single-cell technologies provided new opportunities to study their heterogeneity and role in humans. The main markers used in the different reports to identify the subsets of stromal cells are displayed in Table 2.

**Table 2.** Thymic stromal cell types and subtypes with identifying markers from single-cell omic studies

Study	Cell type	Subtype	Markers
Park et al., 2020	TEC	cTEC	EPCAM, FOXN1, PSMB11
		mcTEC	EPCAM, DLK2
		mTECI	EPCAM, KRT14
		mTECII	EPCAM, AIRE
		mTECIII	EPCAM, KRT1
		mTECIV	EPCAM, FOXJ1
		TEC(myo)	EPCAM, MYOD, CHRNA1
		TEC(neuro)	EPCAM, NEUROD1, CHGA
	Fibroblast	Fib_1	PDGFRA, COLEC11, ALDH1A2, GDF10
		Fib_2	PDGFRA, FBN1, PI16, SEMA3D
		Fb_cycling	PDGFRA, PCNA
	Vascular smooth muscle cells (VSMC)		PDGFRB, ACTA2, RGS5
	Endothelial	Endo	PECAM1, CDH5
		Lymph	PECAM1, LYVE1
Campinoti et al., 2020	TEC	cTEC I	CCL25, CD274, CFC1, CTSV, FOXN1, KCNIP3, LY75, PRSS16, PSMB11, SCX, SLC46A2, TBATA, TP53AIP1
		cTEC II	
		cTEC III	
		mTEC-Myo I	CD24, HES6, EPCAM, CDH1, CLDN3
		mTEC-Myo II	
		mTEC-Neuro I	
		mTEC-Neuro II	
		mTEC-Diff	
		Common (com)TECEMT	CCL19, CFTR, COL1A1, DAB2, FN1, GLIPR2, HAS2, HGF, KRT14, KRT15, LOXL2, MKI67, MMP2, S100A4, SNAI1, SNAI2, TFCP2L1, TGFB3, TGFBR2, THY1, TP63, TWIST1, ZEB1
		comTEC-Proliferating	
		comTEC-Polykeratins	
		comTEC-Ionocytes	
Bautista et al., 2021	TEC	cTEC^hi^	EPCAM, FOXN1, PSMB11, CCL25, HLA-DQB1
		cTEC^lo^	EPCAM, FOXN1, PSMB11
		Immature TEC-1	EPCAM, KRT8, KRT5, SOCS3, KRT17
		Immature TEC-2	EPCAM, KRT8, KRT5, IGFBP5, SAA1, CDH13
		mTEC^lo^	EPCAM, CLDN4, CCL21, KRT15
		Aire+ mTEC^hi^	EPCAM, SPIB, AIRE, FEZF2
		Corneo-like mTEC	EPCAM, KRT1, IVL
		Neuroendocrine	EPCAM, NEUROD1, BEX1
		Myoid	EPCAM, MYOD1, DES
		Myelin+	EPCAM, SOX10, MPZ
	Mesenchyme		PDGFRA, LUM, LAMA2
	Pericytes		PDGFRB, MCAM, CPSG4, FST
	Endothelial	Endo-1	PECAM1, ACKR1, SELE, APLNR, LGALS3
		Endo-2	PECAM1, VEGFC, GJA4
		Endo-3	PECAM1, ACKR1, APLNR, FN1
		Endo-4	LYVE1, PROX1, CCL21
	Mesothelium		MSLN, UPK3B, PRG4
Heimli et al., 2023	TEC	cTEC	EPCAM, PSMB11, PRSS16, CCL25
		NEURL2+ TEC	EPCAM, PRSS16, NEURL2, DLL4
		mcTEC	EPCAM, DLK2, CCL2, KRT15
		mTECI	EPCAM, DLK2, CCL2, KRT15, CCL19, CXCL14
		mTECII	EPCAM, AIRE, HLA-DRA, NTHL1
		mTECIII	EPCAM, IVL, ANXA1, ANXA9
		mTEC(myo)	EPCAM, MYOG, MYOD1, DES
		mTEC(neuro)	EPCAM, NEUROD1, BEX1
		Ciliated	EPCAM, ATOH1, GFI1, LHX3, FOXJ1
		Ionocyte-like	EPCAM, FOXI1,ASCL3, CFTR, CLCNKB
	Fibroblast	Fibro_1	COL15A1, SFRP2
		Fibro_2	IL33, CCL19
		Fibro_3	CCL19, CXCL9, CXCL10, CD40, HLA-DRA
		Fibro_4	DPP4, PI16, SEMA3C, MFAP5,FBN1
	Endothelial	Endo_1	PECAM1, ACKR1, ICAM1, SELE
		Endo_2	PECAM1, ACKR1
		Endo_3	PECAM1, CXCL12, SEMA3G, HEY1, NOTCH4, IL32
		Endo_4	LYVE1, PROX1, FLT4
	Mural	Mural_1	ATF3, NR4A1, CDKN1A, MYH11, TAGLN
		Mural_2	MYH11
		Mural_3	CCL21, CCL19, CXCL12
Ragazzini et al., 2023	TEC	cTEC immediate-early genes	CD274, TBATA, PRSS16, FOXN1, AP-1
		cTEC conventional I–III	TBATA, PRSS16, CTSV, KCNIP3
		cTEC differentiating	TBATA, PRSS16, ATF3, CCL5
		PolyKRT	KRT5, KRT8, KRT13, KRT14, KRT15, KRT17
		PolyKRT proliferating	KRT5, KRT8, KRT13, KRT14, KRT15, KRT17, HMGB2
		mTEC Hassall’s body region	KRT1, KRT6A,KRT10, AIRE, FEZF2
		mTEC Ionocytes	CFTR, TFCP2L1, ASCL3, FOXI1, KRT7
		mTEC differentiating	ASCL1, CLDN3-4
		mTEC Myoid	MYOD, MYOG, TTN, MEFC2
		mTEC Neuro early	NEUROD1, GNG8, SOX11, NKX6-2, MGP, AVP
		mTEC Neuro intermediate	NEUROD1, GNG8, POUF4F1, HIGD1B, GKAP1
		mTEC Mechano high	S100A1, POUF4F3, IRX2, SOX2
		mTEC Mechano low	S100A1, POUF4F3, IRX2, ATOH1
Stankiewicz et al., 2023, *Preprint*	TEC	cTEC	PSMB11, KRT5, KRT8
		mTEC	EPCAM, KRT5, KRT8
		Activated mTEC	EPCAM, CLASS I, CLASS II
		Specialty TEC	EPCAM, NEUROD1, MYOG, POU4F1
	Fibroblast	DPP4+ capsular (cap) Fibs	PDGFRA, PI16, DPP4
		capFibs	PDGFRA, PI16
		Medullary Fibs	PDGFRA, CCL19, CLASS I
		Fibs proliferating	PDGFRA, MKI67
		KRT+ Fibs	PDGFRA, KRT5, KRT8
	VSMC		MCAM, ACTA2
	Pericytes		MCAM
	Endothelial	ECs (Notch)	CD31, JAG1, JAG2, DLL1, DLL4
		ECs	CD31
		Lymphatic endothelial cells	CD31, LYVE1
Yayon et al., 2023, *Preprint*	TEC	cTECI	PSMB11, LY75, CCL25, HLA-DRA
		cTECII	PSMB11, LY75, CCL25, HLA-DRA, DLL4, FOXN1
		cTECIII	PSMB11, LY75, CCL25, HLA-DRA, TBATA, TP53AIP1
		mcTEC	DLK2, IGFBP5, IGFBP6, CCN2, CCL2, ITGA6, KRT15
		mcTEC-Prolif	DLK2, IGFBP5, IGFBP6, CCN2, CCL2, ITGA6, MKI67
		mTECI	EPCAM, ASCL1, CCL21
		mTECI-trans	EPCAM, ASCL1
		mTECII	EPCAM, AIRE, FEZF2, CRIP1
		mTECIII	EPCAM, SLPI, IVL, KRT10, CDKN2A
		TEC-neuro	EPCAM, NEUROD1, BEX1, NEUROG1, NEUROD4
		TEC-ciliated	EPCAM, NEUROD1, BEX1, PCP4, FOXJ1
		TEC-myo	EPCAM, MYOG, TTN, CHRNA1
		TEC-tuft	EPCAM, FOXI1, CFTR, POU2F3, PLCB2
	Fibroblast	PeriloFb	PDGFRA, PDGFRB, VIM, COLEC11, ALDH1A2, GDF10
		PeriloFb-Prolif	PDGFRA, PDGFRB, VIM, COLEC11, ALDH1A2, GDF10, MKI67
		InterloFb	PDGFRA, PDGFRB, VIM, FBN1, PI16, MFAP5, SEMA3C
		InterloFb-COL9A3	PDGFRA, PDGFRB, VIM, FBN1, PI16, MFAP5, SEMA3C, COL9A3, COL13A1, WIF1, DHRS3
		Medullary fibroblasts (medFb)	PDGFRA, PDGFRB, VIM, CCL19, IL33, TNSF10, CXCL9, CXCL10
		medFb-RGS5	PDGFRA, PDGFRB, VIM, CCL19, IL33, TNSF10, CXCL9, CXCL10, RGS5
		medFB-MHCIIh	PDGFRA, PDGFRB, VIM, CCL19, IL33, TNSF10, CXCL9, CXCL10, HLA-DRA, SBSPON, HEY1, LTBP1
		fetFB-CCL21	PDGFRA, PDGFRB, VIM, CCL21, FDCSP, CXCL13
		fetFB-NKX2-5	PDGFRA, PDGFRB, VIM, TNNT1, NKX2-5, KRT8
		fetFB-RSPO2	PDGFRA, PDGFRB, VIM, CES1, RSPO2
	Endothelial	EC-Art	PECAM1, CDH5, CLDN5, CXCL12, SEMA3G, HEY1
		EC-Art-ELN	PECAM1, CDH5, CLDN5, SULF1, ELN, CXCL12, SEMA3G, HEY1
		EC-Cap	PECAM1, CDH5, CLDN5, RGCC, PLVAP
		EC-Cap-Prolif	PECAM1, CDH5, CLDN5, RGCC, PLVAP, MKI67
		EC-Ven	PECAM1, CDH5, CLDN5, PLVAP, ACKR1, ICAM1, CCL2, SELE
		EC-Ven-ELN	PECAM1, CDH5, SULF1, ELN, PLVAP, ACKR1, ICAM1, CCL2, SELE
		EC-Lymphatic	CLDN5, PROX1, TFF3, CCL21
	Pericytes	SMC	ACTA2, MYH11, RERGL, CASQ2, ELN
		Pericyte	RGS5, PDGFRB, ABCC9, KCNJ8
		Pericyte_CCL19	RGS5, PDGFRB, ABCC9, KCNJ8, CCL19, CCL21
		Pericyte_COL1A1	ACTA2, COL1A1, ELN
		ProlifPericyte	ACTA2, RGS5, PDGFRB, ABCC9, KCNJ8, MKI67, TOP2A

#### TEC progenitors

In a very recent report, Ragazzini and colleagues took on the complex task of trying to identify TECs with stem cell properties in human postnatal thymus by using a combination of high-resolution in vitro and in vivo scRNA-seq on isolated cells, clonal expansion, and differentiation assays (Ragazzini et al., 2023). To achieve this goal, they performed scRNA-seq separately on sorted cortical TECs (cTECs) (EpCAM^low^CD205^+^) and mTECs (EpCAM^high^CD205^−^) and identified 16 clusters. With this strategy, they were able to identify four clusters of cTEC, three of which were previously described (cTEC I–III) (Bautista et al., 2021; Campinoti et al., 2020; Park et al., 2020), and will be discussed later more in detail, while the fourth one was unique to this study and was characterized by high expression of immediate-early genes (e.g., *JUN*, *FOS*, and *ATF3*) and *CD274*, in addition to cTEC-specific genes (e.g., *FOXN1*, *TBATA*, and *PRSS16*). They also identified seven specialized mTEC clusters, which included subsets previously known, such as myoid, ionocytes, corneocytes, and neuroendocrine cells. Most importantly, they identified a cluster of putative epithelial stem cells, which was common between the cTEC and mTEC sorted populations and presented a distinctive signature, characterized by expression of genes encoding for extracellular matrix (ECM) proteins, molecules anchoring to ECM (e.g., *FN1*, *BCAM*, and *VCAM1*), and multiple keratins, including some that in other tissues have been associated to proliferating stem cells (e.g., *KRT15*) or differentiated layers (e.g., *KRT13*). These cells were thus called PolyKRT, were found to localize at sub-capsular and perivascular spaces, and showed stem cell properties and multilineage differentiation capabilities when isolated and expanded in vitro (Ragazzini et al., 2023).

#### Novel subsets of cTEC and mTEC

To improve the characterization of thymic stromal cells in the Human Thymus Atlas, Park et al. performed scRNA-seq on EPCAM-enriched samples encompassing fetal, early postnatal, and adult human thymus samples (Park et al., 2020). In line with previous work on murine thymus (Baran-Gale et al., 2020; Bornstein et al., 2018; Givony et al., 2023; Kernfeld et al., 2018; Michelson et al., 2022, 2023; Miller et al., 2018), TECs could be divided into multiple subtypes, with mTECs showing the largest degree of heterogeneity. *PSMB11*-positive cTECs, keratin *(KRT)14*–positive mTECs (mTECI), autoimmune regulator (*AIRE*)–expressing mTECII, and *KRT1*-expressing mTECIII could be identified. Notably, cTECs are more prevalent during early developmental stages (7–8 post-conception weeks), while an intermediate population termed mcTECs, characterized by *DLK2* expression, emerged during late fetal and pediatric stages. Additionally, a rare population of mTECIV resembling tuft-like cells in humans was identified. However, markers like *DCLK1* or *POU2F3*, commonly used to define mTECIV in mice (Bornstein et al., 2018; Miller et al., 2018), exhibited enrichment but lacked specificity in human samples. Furthermore, two distinct EPCAM^+^ cell types were recognized for the first time in humans, and later on identified also in mice (Michelson et al., 2022): *MYOD1*- and *MYOG*-expressing myoid cells (TEC[myo]), and *NEUROD1*-, *NEUROG1*-, and *CHGA*-expressing TEC(neuro), reminiscent of neuroendocrine cells (Park et al., 2020) (see Table 2 for details).

The study by Park and colleagues allowed us to obtain an unprecedented knowledge of the complexity of the thymic stromal compartment. Yet, even if the authors enriched for EpCAM^+^CD45^−^ cells in some of their samples, the number of stromal cells included in their manuscript was still relatively limited. For this reason, the contemporary reports by Bautista and colleagues (Bautista et al., 2021) and Campinoti and colleagues (Campinoti et al., 2020), which focused on the characterization of the stromal compartment, allowed further significant progress in the characterization of human thymus stromal cells (Table 2). The dataset by Bautista and colleagues included tissues from two fetal, two postnatal, and one adult (25 years old) individuals, which were enzymatically dissociated and depleted of CD45^+^ hematopoietic cells. While TECs were confirmed to be the most abundant stromal compartment, this work highlighted the critical role played by other stromal cell types, which will be discussed more in detail below. A particular focus of this work was to clearly delineate the heterogeneity of human TECs and understand their lineage progression from fetal to adult life. To this purpose, TECs were reanalyzed independently from the other stromal populations and reclustered based on their gene expression profiles (Fig. 1 D, left). This allowed to identify two cTEC populations (cTEC^hi^ and cTEC^lo^), distinguished by different levels of cTEC markers and Notch signaling activity. Pseudotime analysis suggested that cTEC^lo^ gives rise to cTEC^hi^ in both fetal and postnatal tissues. Furthermore, cTEC^hi^ cells were observed to decrease in number with time. Bautista et al. (2021) also identified two populations of mTECs expressing *KRT15* and low levels of MHC molecules (immature TEC and mTEC^lo^). Immature TECs are a particularly intriguing population, expressing canonical TEC identity genes (*FOXN1*, *PAX9*, *SIX1*) but lacking the expression of functional genes of cTECs or mTECs, and could possibly represent progenitors or cells that have lost their differentiated phenotype. In support of the latter hypothesis, a subpopulation of immature TEC-2 cells has been identified, which is marked by the expression of factors involved in thymic involution (*CDH13* and *IGFBP5*, among others), and accumulates in older tissue to the detriment of functional TECs. A recent study in which thymic stroma and thymocyte datasets were merged and computational tools were used to explore cell–cell communication patterns and gene regulatory networks over time identified IGFBP5 as a marker of thymic involution and a target of specific regulons that induce epithelial-to-mesenchymal transition (EMT), such as *FOXC1*, *KLF9*, and *MXI1* (Yang et al., 2024). In vitro functional assays showed that when *IGFBP5* is overexpressed in TECs, thymocyte proliferation is inhibited, opening new perspectives to understand molecular mechanisms of thymic aging and involution.

Furthermore, the study of Bautista and colleagues demonstrated that AIRE-expressing TECs and additional subsets of mTECs (ionocytes, ciliated, neuroendocrine, and myoid cells) play distinct and complementary roles in tolerance induction (Bautista et al., 2021). While AIRE^+^ TECs express a wide array of tissue-specific antigens (TSA), the other mTECs have a more restricted TSA expression profile targeting selective peripheral tissue antigens. For instance, they propose a role for myoid TECs in tolerance induction to muscle antigens (Bautista et al., 2021).

The fine annotation of thymic stromal cells developed by Park and Bautista was subsequently confirmed by Heimli and colleagues, who also identified a cTEC cluster termed NEURL2^+^, defined by expression of *NEURL2* and *DLL4*, which showed higher resemblance to mcTECs compared with other cTECs (Heimli et al., 2023) (Table 2). In this study, thymic epithelial cells were predicted to engage in interactions with specific thymocyte populations. The mcTEC/mTECI cluster was suggested to interact with thymocytes via CCL19, while the mTECII cluster was implicated in interactions through CCL17.

#### Non-epithelial stromal cells

The importance of non-epithelial stromal cells in the thymus has received broader recognition in recent years (Foster et al., 2008; Gordon and Manley, 2011; Manley et al., 2011; Nitta and Takayanagi, 2021). For this reason, scRNA-seq–based studies of the human thymus have paid special attention to the characterization of non-epithelial cells within the stromal cell compartment (Fig. 1 E, left). Park and colleagues in their dataset (Park et al., 2020) defined two fibroblast subtypes (named fibroblast type 1 [Fb1] cells and fibroblast type 2 [Fb2] cells) that were characterized by divergent patterns during development. Fb1 (*COLEC11*^+^, *C7*^+^, *GDF10*^+^) expressed factors important for epithelial cell development, such as enzymes responsible for the production of retinoic acid, and dominated during early development, while similar numbers of Fb1 and Fb2 (*PI16*^+^, *FN1*^+^, *FBN1*^+^) cells were observed later in development. In the report by Bautista and colleagues (Bautista et al., 2021), mesenchymal cells were also found to express a variety of ligands and regulators of critical pathways involved in the development and function of TECs, such as BMP4, FGF7, and Wingless-related integration site (WNT) ligands, especially in fetal and early postnatal samples. The authors also identified pericytes and hypothesized that these cells might contribute to TEC differentiation by expressing Activin A subunits, while its antagonist follistatin (FST), which promotes TEC progenitor maintenance, is mostly expressed by adult mesenchymal cells. Heimli and colleagues added to the complexity of the fibroblast subsets by identifying four clusters, each with distinct expression patterns suggesting immune interaction or specific tissue roles, and with capsular or medullary localization (Heimli et al., 2023). In this study, they also identified mural cells that were grouped into three clusters, with one showing a contractile phenotype and another expressing specific chemokines, including *CCL21*, *CCL19*, and *CXCL12*.

Finally, all these reports characterized several subsets of endothelial cells expressing extracellular matrix, adhesion molecules, and chemokines important for thymocyte migration. For details on the subsets identified in each study and the markers used, refer to Table 2.

#### Does sex matter?

A recent report by Stankiewicz and colleagues (Stankiewicz et al., 2023, *Preprint*) has provided evidence that sex differences might affect composition and transcriptional profile of human thymus cells (Fig. 1 B, right). By analyzing six postnatal thymic samples of the same age (4 months), evenly divided between males and females, these authors observed several gene expression signatures that were consistently upregulated in male or female cells across multiple cell types, with female cells showing higher expression levels of genes involved in metabolism, translation, and antigen presentation, while male cells had increased expression of genes involved in adipogenesis, proinflammatory signaling, and ECM pathways. For other pathways, sex-based enrichments were detected in only a few cell types. For instance, higher cytokine signaling was detected in female T cells and hematopoietic cells, while in male cells this signature was found in epithelial and mesenchymal cells. Additionally, differences were observed in cell type abundance between male and female samples, with an increase in DP and cTECs in females and in SP, CD3^+^ DPs, and mTECs in males. In addition, sex differences were detected in the Notch–ligand interactions in the early development of ETPs in the cortico-medullary junction niche, with JAG ligand interactions being more abundant and diverse in females than in males, which could lead to alternative lineage development pathways. Several subsets of fibroblasts (DPP4^+^ fibroblasts, capsular fibroblasts, and a novel subset of proliferating fibroblasts) were identified and mapped to the subcapsular niche. DPP4^+^ fibroblasts were the main responders to sex hormones and could be implicated in the sex hormone–associated thymic involution. Capsular fibroblasts expressed genes involved in cytokine and chemokine signaling and might play a role in thymocyte migration, while proliferating fibroblasts were characterized by upregulation of WNT signaling genes and cell sensing pathways. Sex differences were also observed in fibroblast growth factor (FGF) signaling in fibroblasts, with increased expression of the cTEC growth factor *FGF7* in male samples that could explain the larger size of early postnatal thymi in males. Interestingly, expression of *APOD*, a gene associated with increased adipogenesis, was also detected in male fibroblasts, which could contribute to earlier initiation of thymic involution in males.

This report proposes interesting hypotheses on how sex may induce differences in thymic development, functionality, and involution in humans. However, the cohort analyzed was small (three samples each from females and males), and of the same age (4 months). Studies including a larger number of thymus samples from subjects of various ages will be needed to confirm these hypotheses.

## Spatial transcriptomic and multiplex imaging: Location matters

While scRNA-seq has allowed to gain enormous knowledge on the diversity of the populations residing in the thymus, understanding the complex cellular interactions inside the human thymus, both in qualitative and spatial terms, has been extremely challenging if not impossible for many years due to the lack of high-resolution spatial systems. However, in the last couple of years, the application of multidimensional imaging techniques and spatial transcriptomics, in combination with single-cell transcriptomics, has allowed to gain better understanding of the thymic niches and provided novel insights into the biology of human thymus. However, it has to be pointed out that while several approaches of spatial transcriptomics reaching single-cell resolution are currently available (Chen et al., 2023), the studies reported so far on the human thymus have all been performed using the 10X Genomics Visium spatial transcriptomic technique. This approach has a resolution at a spot of 55 μm so that each spot most likely contains multiple heterogeneous cells. For this reason, to define the cells inside each of the 55 μm spots, a bioinformatic approach called deconvolution needs to be used (Williams et al., 2022). The spot deconvolution aims at untangling the transcriptomic profiles coming from each of the cells contained in the spot. To assign to each cell their specific gene expression profile, reference datasets are applied, which are derived from scRNA-seq in which the individual tissue-specific cell types have been annotated. Several deconvolution approaches have been developed for spatial transcriptomics analysis, and each of them presents advantages and limitations (Kleino et al., 2022; Zhang et al., 2022).

In the context of extensive mapping of the developing immune system across several organs, Suo and colleagues (Suo et al., 2022) combined scRNA-seq and spatial transcriptomics in samples isolated in prenatal life from nine organs, including hematopoietic (yolk sac, liver, and bone marrow), lymphoid (thymus, spleen, and lymph node), and non-lymphoid organs (skin, kidney, and gut). In this report, spatial transcriptomics was performed in two thymic samples, at 16 and 18 wk after conception, and was mainly employed to define cellular localization within known histological structures. A larger cohort of thymic samples analyzed by spatial transcriptomics was published a few months later and included eight samples, collected in postnatal life, between 10 days and 1 year after birth (Heimli et al., 2023). The authors performed manual annotation of the clusters, using both transcriptomic profiles and spatial localization, and identified seven clusters that were divided mainly based on their localization in the cortex, medulla, cortico-medullary junction, and interlobular zones. They also identified a cluster with high expression of hemoglobin genes (“hemoglobin rich”) and four clusters with high expression of inflammatory genes: three of them were located at the interlobular region and were named “inflammatory” and one (named “Hassall associated”) was located in the medulla and expressed also corneocyte genes. In the study by Heimli et al., deconvolution was performed to identify the cell types represented in each of the spots, generating 31 clusters. As expected, cortical spots were mainly formed by DP and cTECs, while the medulla showed higher heterogeneity of cell composition, and included SP, mTECs, B cells, and DC. This study also showed that DCs are located not only in the medulla, where they contribute to agonist selection, but also at the cortico-medullary junction. Similar findings had been reported by Suo and colleagues in prenatal samples (Suo et al., 2022), supporting the reproducibility of results across separate cohorts. Finally, Heimli et al. identified different subsets of fibroblasts with medullary and cortical localization, thus confirming earlier reports using scRNA-seq of fibroblast subsets with distinct transcriptional profiles and functions correlating with their localization (Bautista et al., 2021; Park et al., 2020).

An alternative approach to generate a map of thymic niches in postnatal samples, based on the combination of ATAC-Seq, CITE-seq, and a multidimensional imaging platform CO-detection by-indEXing (CODEX) has been proposed by Stankiewicz et al., mentioned earlier for their provocative results on sex differences in human thymus (Stankiewicz et al., 2023, *Preprint*). Using the combination of these approaches, Stankiewicz et al. characterized the medullary area surrounding the HCs and identified several niches involved in negative selection, cross-presentation, and mTEC maturation (Stankiewicz et al., 2023, *Preprint*) (see Table 2 for details on stromal cell subsets).

Yayon, Kedlian, Boehme, and colleagues took an even more comprehensive approach to spatial mapping of the human thymus (Yayon et al., 2023, *Preprint*) (see Table 2 for details on stromal cell subsets). Their cohort of samples contained prenatal and postnatal samples, ranging from post-conception week 11 to 3 years of age. A combination of scRNA-seq, CITE-seq, and scTCR-seq was used to analyze dissociated cells, along with three complementary spatial technologies: spatial transcriptomics, protein imaging using a 44-plex Iterative Bleaching Extends multipleXity (IBEX) panel (Radtke et al., 2022), and 14-plex RareCyte panel (see Table 1 for details on the techniques used in this report). One of the main achievements of this study has been the generation of a continuous common coordinate framework, allowing the establishment of consistent structural annotations that could be used to compare samples from different cohorts and analyzed with different techniques. To achieve this goal, the three major histological structures of the thymus (capsule, cortex, and medulla) were used as anchor points to generate the cortico–medullary axis (CMA), along which each cell in the thymus can be uniquely mapped based on their distance from a structural landmark. Each Visium spot from fetal and postnatal samples was then mapped to the CMA (Fig. 1 A, right). To uniquely annotate cells across samples, single-cell data from fetal and postnatal samples were integrated to generate common cell annotations, and annotated cells were then mapped to 10X Genomics Visium spatial transcriptomics datasets after spot deconvolution with cell2localization (Kleshchevnikov et al., 2022). The thymus map generated was used to study conventional T cell differentiation across development, revealing a largely conserved spatial trajectory for the development of αβT cells in fetal and pediatric samples. The distribution of the major cytokines and chemokines expressed in the thymus and involved in the migration of developing T cells showed similar distribution across the CMA in fetal and postnatal samples. However, some cytokines (IL-34, SPP1, and IL1R1 and IL1R2) showed distinct spatial localization across development, suggesting that they may play a different role in pre- and postnatal thymus. While some cell types (such as capsular and sub-capsular fibroblasts) maintained a similar spatial distribution in pre- and postnatal thymi, medullary fibroblasts were predicted to reside all in the medulla in pediatric samples, whereas only a subset of them had a medullary localization in fetal samples (Fig. 1 E, right). These differences may imply migration and/or maturation during development. In the TEC compartment, the niche of the putative TEC progenitors, the medullary cortical TEC (mcTEC), was mapped to different spatial localizations in fetal and pediatric samples: mcTEC mapped closer to the capsule in fetal samples and closer to the cortico–medullary junction and perivascular region in postnatal samples, confirming previous observations (Ragazzini et al., 2023). Moreover, Yayon et al. found that the cells more closely associated with HCs were the highly differentiated mTECIII cells (Yayon et al., 2023, *Preprint*). These cells were found to express high levels of tissue-restricted antigens, indicating an important role of HCs niches in the development of central tolerance (Fig. 1 C, right). Finally, a differential migration pattern was observed for T cells developing towards the CD8 and CD4 lineages. In particular, CD8 cells stayed longer in the cortex and moved to the medulla only as they reached the final stage of maturation, while CD4 cells migrated to the medulla already at a semi-mature stage (Fig. 1 D, right). These differential migratory pathways were attributed to differences in the pattern of expression of CCR4 and CXCR3, the chemokine receptors driving the migration of CD4 and CD8 cells, respectively, toward the medulla. Overall, generation of the continuous CMA may provide the scientific community with a tool to allow harmonization of data obtained through the analysis of additional cohorts of samples and with multiple techniques.

## Organoid systems: Recreating in vitro the thymus niches

Even though the technological advances described above have allowed major progress in understanding human thymus biology, several challenges remain to study human thymus development and function, in particular in pathological conditions. Access to human thymus is very limited since the tissue is typically obtained when partial or total thymectomy is performed during cardiothoracic surgery or from postmortem autopsies. In addition, complete athymia is associated with the most severe genetic defects that affect thymus development. For these reasons, there is still the need to generate in vitro models, using human cells, that faithfully recapitulate the early stages of thymic stromal and hematopoietic cell development and function, both in health and disease. In the last few years, important advancements have been made in the development of protocols for the in vitro differentiation of human thymic epithelial and hematopoietic cell progenitors and in the generation of human cell-based thymic organoids.

For many years, the most widely used technique to model in vitro T cell development was the one developed by the group of Zúñiga-Pflücker, which involved coculturing in 2D hematopoietic stem and progenitor cells (HSPCs) with the murine stromal cell line OP9 engineered to express the Notch ligand-Delta like 1 (DL1) or -Delta like 4 (DL4) (Schmitt and Zúñiga-Pflücker, 2002, 2006). This technique was also adapted to replace HSPCs with induced pluripotent stem cells (iPSCs) (Holmes and Zúñiga-Pflücker, 2009). More recently, the group of Gay Crooks developed a 3D system, called artificial thymic organoid (ATO) assay, based on coculturing the murine stromal cell line MS5 engineered to express DL1 or DL4 with HSPCs in serum-free medium (Seet et al., 2017). This assay proved to be more efficient in generating mature T cells from CD34^+^ cells isolated from bone marrow, cord blood, and mobilized peripheral blood of normal donors (Seet et al., 2017). The group of Gay Crooks also demonstrated how the transcriptional metabolic patterns and metabolic extracellular flux results in ATO-derived T cells were largely similar to those of ex vivo–isolated thymocytes (Sun et al., 2021).

Montel-Hagen and colleagues were also able to adapt the ATO protocol to differentiate iPSCs into mature T cells (Montel-Hagen et al., 2019). Generation of large amounts of T cells from iPSC lines could have important value for clinical applications, such as production of chimeric antigen receptor (CAR) T cells for use in cancer immunotherapy (Montel-Hagen and Crooks, 2019). Of note, a recent report showed that depending on the strength of the CAR, iPSCs transduced with a CD19-targeted CAR and then differentiated in the ATO platform may manifest diversion of T cell differentiation from conventional T cell lineages to the ILC2 lineage (Li et al., 2023).

We have demonstrated that the ATO platform can be very useful in modeling in vitro faulty T cell development of CD34^+^ cells from patients with known hematopoietic-intrinsic gene defects associated with severe combined immune deficiency (Bosticardo et al., 2020). Using a similar approach, Bifsha and colleagues developed a 3D system in which OP9-DL4 cells were co-cultured with CD34^+^ cells isolated from unmobilized peripheral blood (Bifsha et al., 2020). Both systems reliably distinguished hematopoietic cell–autonomous versus thymus-intrinsic defects of T cell development and could potentially be used to help define the etiology of severe congenital T cell lymphopenia and prompt appropriate definitive therapy (hematopoietic stem cell transplantation versus thymus implantation).

Moreover, the ATO system has allowed to better characterize novel forms of T cell deficiency in which access to thymus would have otherwise been impossible (Delmonte et al., 2021; Gazzin et al., 2023; Ghosh et al., 2022; Nichols-Vinueza et al., 2021; Yakici et al., 2023). Most recently, the ATOs have been used to evaluate the T cell differentiation potential of CD34^+^ cells from patients with mutations in the *PTCRA* gene (Materna et al., 2024). ATOs generated with *PTCRA*-mutant cells showed a selective defect in TCRαβ^+^ cell production, directly correlating with the severity of their genetic mutation, while the ability to make TCRγδ^+^ cells was preserved (Materna et al., 2024). Additionally, ATOs can be used to evaluate the effect of mutations induced in normal CD34^+^ cells, as in a recent publication in which CRISPR/Cas9 was used to knock out the *WAS* gene in human hematopoietic stem cells (Pille et al., 2023). *WAS* KO CD34^+^ cells showed a partial block in the generation of CD8^+^ SP cells (Pille et al., 2023). In another report, ATOs were used to evaluate the effects of purine nucleoside phosphorylase (PNP) inactivation, an enzyme mediating the breakdown and recycling of guanine nucleoside, whose deficiency in humans is associated with both immunodeficiency and autoimmunity (Al-Saud et al., 2020; Giblett et al., 1975; Markert, 1991). When PNP function was inactivated in the ATOs using a specific inhibitor, the CD34^+^ cells had decreased viability and accumulated at the DN stage. These effects were proved to be mediated through the downregulation of the deoxynucleoside triphosphate (dNTP) triphosphohydrolase SAMHD1 (Abt et al., 2022). The ATO platform can also be used to better define cellular mechanisms involved in the positive selection of various T cell types. In particular, Suo et al. used the ATO system to provide experimental evidence that PLZF-expressing unconventional T cells are positively selected upon interaction with other T cells, as opposed to conventional T cells, which are selected through signals provided by cTECs (Suo et al., 2022). More recently, Frenz-Wiessner et al., 2024) employed the ATO system to prove the T cell differentiation potential of bone marrow-like organoid (BMO)–derived hematopoietic progenitors.

**Figure 2.**
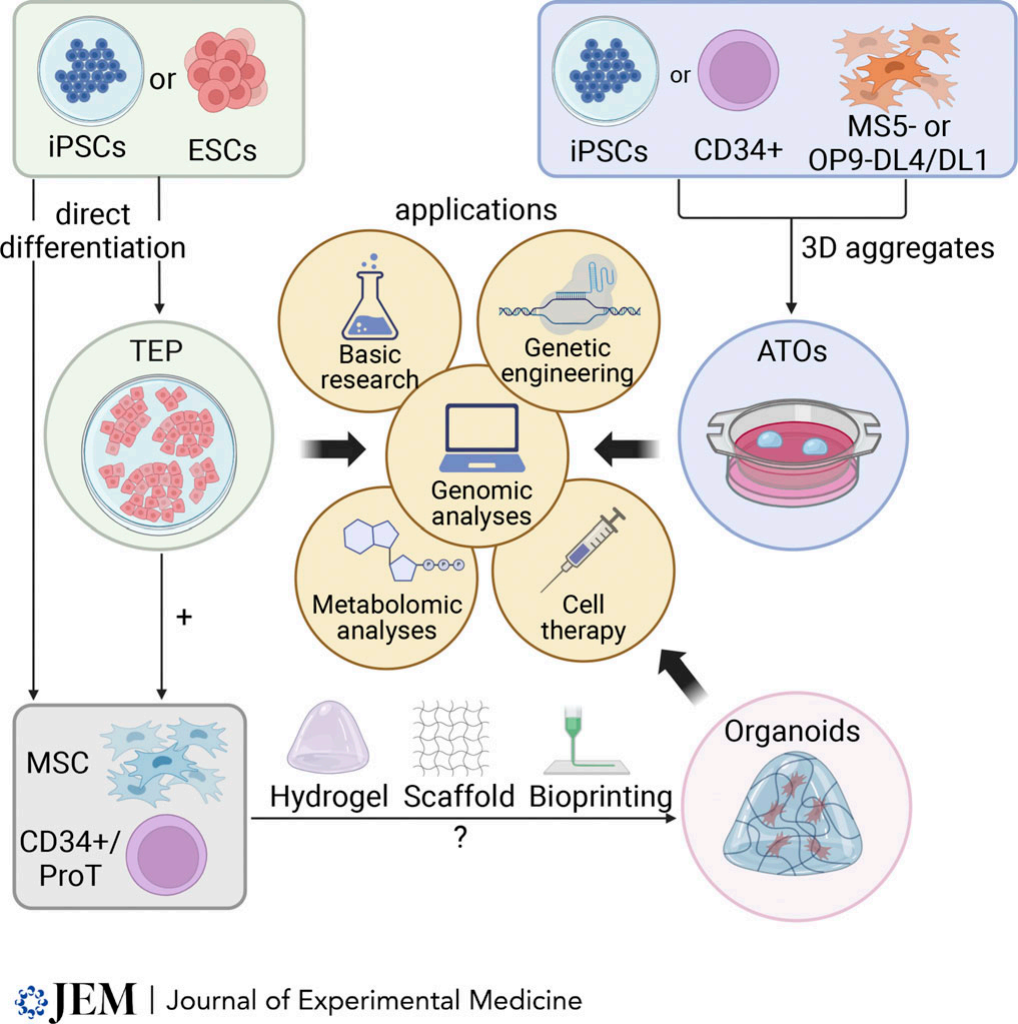
**In vitro modeling of T cell and stromal cell development and potential applications.** The figure shows that TEP, mesenchymal cells, and CD34/ProT cells can be generated by direct differentiation from iPSCs or ESCs, while mature T cells can be obtained using 3D aggregation of iPSCs or CD34^+^ cells with stromal cell lines expressing Notch ligands (MS5- or OP9-DL4/DL1). A combination of hematopoietic progenitors, mesenchymal cells, and epithelial cells in 3D structures that can be based on hydrogel, scaffolds, or bioprinted materials are being tested to create all-human thymi in a dish. All these models can be used to perform a variety of studies and could be the base for the development of novel therapeutic approaches.

Finally, the ATO assay has proven extremely useful in the preclinical validation of gene and base editing approaches in CD34^+^ cells from patients carrying genetic mutations that severely compromise T cell development and/or function, such as mutations in the IL2 receptor common γ chain (*IL2RG*) (Brault et al., 2023), recombinase activating gene 2 (*RAG2*) (Gardner et al., 2021), *RAG1* (Castiello et al., 2024), *CD3D* (McAuley et al., 2023), and *MAGT1* (Brault et al., 2021) genes. Altogether, these reports (summarized in Table 3 and Fig. 2) highlight the versatility and reliability of the ATO assay, which has allowed to uncover new knowledge on early T cell development in health and disease and has provided an efficient means to test the preclinical efficacy of novel therapeutic interventions. However, caution must be applied when interpreting results of ATOs for clinical diagnosis. While interpretation of the results is more straightforward when the ATOs show an early developmental block, the successful differentiation to mature T cells might not conclusively rule out a hematopoietic cell–autonomous defect. In particular, the ATO system does not allow to identify forms of severe T cell deficiency due to intrathymic migration defects nor late defects of T cell development (beyond the DP stage) or SCID due to adenosine deaminase (ADA) deficiency. In the latter, detoxification provided in trans by the MS5 or OP9 stromal cells allows survival and differentiation of ADA-deficient HSPCs (Bifsha et al., 2020; Bosticardo et al., 2020).

**Table 3.** Summary of published reports using 3D organoids for in vitro T cell differentiation

Gene defect	Starting cells	Use of the 3D organoids	Results	Reference
Healthy donor (HD)	CD34^+^	Original protocol for the generation of ATOs starting from human CD34^+^ cells	Establishment of a protocol for the efficient in vitro generation of mature T cells, fully functional and with a diverse TCR repertoire	Seet et al., 2017
HD	iPSCs, embryonic stem cells	Original protocol for the generation of ATOs starting from human iPSCs and ES	Establishment of a protocol for the efficient in vitro generation of mature T cells, fully functional and with a diverse TCR repertoire	Montel-Hagen et al., 2019
*AK2*, *IL2RG*, DGS, *RAG1/2*, *ADA*	CD34^+^	Test ATOs to evaluate block in differentiation in patients with known gene defects	(1) Definition of T cell developmental block in patients with hematopoietic-intrinsic defects; (2) discrimination between hematopoietic-intrinsic and -extrinsic defects	Bosticardo et al., 2020
*IL2RG*, *TBX1*, DGS, *RAG1/2*, *ADA*	CD34^+^	Test 3D aggregates to evaluate block in differentiation in patients with known gene defects	(1) Definition of T cell developmental block in patients with hematopoietic-intrinsic defects; (2) discrimination between hematopoietic-intrinsic and -extrinsic defects	Bifsha et al., 2020
*POLD1*	CD34^+^	Evaluation of T cell differentiation in vitro	POLD1-mutant CD34^+^ cells showed a block in differentiation, resulting in a reduced number of CD4^+^CD8^+^ DP cells and almost complete absence of TCRαβ^+^CD3^+^ cells	Nichols-Vinueza et al., 2021
*RAG2*	iPSCs	Test efficacy of CRISPR/Cas9-mediated gene editing (GE) approach	GE-iPSCs of a RAG2-mutant patient overcome block at CD4^+^CD8^+^ DP stage and show normal differentiation to mature TCRαβ^+^CD3^+^ cells	Gardner et al., 2021
*SASH3*	CD34^+^	Evaluation of T cell differentiation in vitro	*SASH3*-mutant CD34^+^ cells were able reach full maturation, but frequency and absolute count of TCRαβ^+^CD3^+^ cells were significantly decreased, due to impaired cell cycle progression and increased apoptosis	Delmonte et al., 2021
*MAGT1*	CD34^+^	Test efficacy of CRISPR/Cas9-mediated GE approach	(1) GE CD34^+^ cells from XMEN patients showed full T maturation; (2) restoration of NKG2D expression in CD3^+^ cells	Brault et al., 2021
HD	CD34^+^	Understanding how PNP inactivation affects T cell differentiation from iPSCs	(1) PNP inactivation causes decreased viability of CD34^+^ cells and accumulation in the CD4^−^CD8^−^ DN stage; (2) effects of PNP inactivation were mediated through the downregulation of the dNTP tri-phosphohydrolase SAMHD1	Abt et al., 2022
HD	iPSCs	Test hypothesis for T-T–mediated selection of unconventional T cells	PLZF-expressing unconventional T cells originate from positive selection on other T cells, as opposed to conventional T cells, which are selected by cTECs	Suo et al., 2022
*FOXI3*	CD34^+^	Evaluation of T cell differentiation in vitro	(1) CD34^+^ cells isolated from a patient with *FOXI3* haploinsufficiency show efficient differentiation to mature TCRαβ^+^CD3^+^ cells; (2) these results support the thymic-intrinsic nature of T cell lymphopenia in *FOXI3*-mutant patients	Ghosh et al., 2022
HD	CD34^+^, TSP	Evaluation the T cell differentiation potential of the different TSP (TSP1, TSP2, TSP3)	TSP1 are a more quiescent population; TSP3 cells are primed to develop much more rapidly into T cells compared with TSP1 and TSP2 cells	Cordes et al., 2022
*IL2RG*	CD34^+^	Test efficacy of lentiviral vector– and CRISPR/Cas9-mediated GE approaches	Both treatments overcome the block at the CD34^+^ Pro T stage and show restoration of surface expression of IL2Rgc and its mediated signaling	Brault et al., 2023
*TRIM37*	CD34^+^	Evaluation of T cell differentiation in vitro	Normal mature TCRαβ^+^CD3^+^ cell generation, excluding an early hematopoietic-intrinsic defect of T cell development	Gazzin et al., 2023
*CD3D*	CD34^+^	Test efficacy of CRISPR/Cas9-mediated BE approach	Edited *CD3D*-mutant patients’ CD34^+^ cells differentiated to fully mature T cells, displaying diverse TCR repertoires and TCR-dependent functions	McAuley et al., 2023
HD	iPSCs	Understanding how CARs affect T cell differentiation from iPSCs	iPSCs transduced with a CD19-targeted CAR showed a diversion of T cell differentiation from conventional T cell to ILC2 lineage	Li et al., 2023
*WAS*	CD34^+^	Evaluation of T cell differentiation in vitro	(1) Partial block at the positive selection checkpoint, especially on the generation of CD8^+^ SP cells; (2) no effects on the generation of a diverse TCR repertoire	Pille et al., 2023
*PAX1*	CD34^+^	Evaluation of T cell differentiation in vitro	Normal mature TCRαβ^+^CD3^+^ cells generation, excluding an early hematopoietic-intrinsic defect of T cell development	Yakici et al., 2023
*RAG1*	CD34^+^	Test efficacy of CRISPR/Cas9-mediated GE approach	(1) Integration into intron 1 of RAG1 gene achieved suboptimal correction and didn't overcome the block in differentiation at the CD4^+^CD8^+^ DP stage; (2) in-frame insertion into exon 2 led to efficient generation of mature TCRαβ^+^CD3^+^ cells	Castiello et al., 2024
HD	iPSCs	Evaluation of the lymphoid differentiation potential of BMO-derived HSPC	BMO-derived HSPCs can differentiate to mature TCRαβ^+^CD3^+^ cells	Frenz-Wiessner et al., 2024
*PTCRA*	CD34^+^	Evaluation of T cell differentiation in vitro	Selective defect in TCRαβ^+^CD3^+^ cell production, directly correlating with the severity of the genetic mutation, while the ability of producing TCRγδ^+^CD3^+^ cells was preserved	Materna et al., 2024

The ATOs represent a great model to study faulty T cell development due to hematopoietic cell–autonomous defects; however, in some instances, the cause of severe congenital T cell lymphopenia is the result of genetic defects affecting the stromal compartment of the thymus, in particular TECs, so that the crosstalk between TECs and developing thymocytes is compromised, leading to impaired maturation of the T cells. In these cases, the patients’ CD34^+^ cells are able to efficiently differentiate in vitro into T cells when tested in the ATO system. Therefore, modeling faulty T cell development due to defects of the thymic stroma requires assays that recapitulate TEC differentiation in vitro. In the last 15 years, many groups have developed protocols for the differentiation of human embryonic stem cells (hESCs) or iPSCs into thymic epithelial progenitors (TEPs) (reviewed in Bosticardo and Notarangelo, 2023; Provin and Giraud, 2022). The use of these protocols has allowed identification of distinctive abnormalities in the transcriptional signature that characterizes TEPs from patients with rare forms of severe T cell lymphopenia of thymic origin, such as *EXTL3* (Volpi et al., 2017) and *PAX1* deficiency (Yamazaki et al., 2020). However, a limitation of these studies is represented by the incomplete maturation of TEPs, which could only differentiate into mature TECs when transplanted in vivo into suitable mouse models, where TEP were provided the environmental cues that are necessary to support their final maturation. Development of novel protocols that would allow differentiation of human iPSCs into mature TECs in vitro would be of fundamental importance to study human TEC biology and to better define the pathophysiology of genetic conditions characterized by functional impairment of TECs. This goal may require generation of organoids in which different cell types are simultaneously present to mimic the complex microenvironment of the developing thymus. Ramos and colleagues (Ramos et al., 2023) combined human iPSC-derived TEP, hematopoietic progenitor cells, and mesenchymal cells to generate functional thymic organoids (TOs). They demonstrated that TOs could support TEC maturation in vitro after 2–4 wk in culture, as shown by the expression of HLA-DR, CD205, KRT5, and AIRE. Importantly, while TEC maturation in the TOs was observed even in the absence of hematopoietic progenitor cells, when the TOs were combined with hematopoietic progenitor cells, they promoted T cell development. To generate TEPs from iPSCs, Provin and colleagues (Provin et al., 2023, *Preprint*) utilized for the first time a protocol generated with an unbiased multifactorial method based on a statistical framework, called optimal design of experiments, which allows to collect the highest amount of information from a limited number of experiments. Using their 2-wk protocol, they could efficiently generate TEP, which were further matured in vitro to cTEC and mTEC through a 3D co-culture system with pro-T cells (Lin^−^CD34^+^CD7^+^) isolated from human thymus and plated on a fibrin-based hydrogel. Finally, Fu and colleagues (Fu et al., 2024) were able to achieve in vitro maturation of TEP to cTECs and mTECs, expressing CD205, HLA-DR, FOXN1, K5, and AIRE, by applying changes to their differentiation protocol, through the combination of high amounts of retinoic acid (1 μM), a TGFβR kinase inhibitor (SB-431542), and WNT3a. Using this new protocol, the mature TECs generated in the system were able to support T cell differentiation in vitro. Interestingly, the authors used this protocol to test the effect on TEC generation and gene expression resulting from the knockdown of *HOXA3*, a transcription factor shown to be essential for the formation of third pharyngeal pouch derivatives like thymus and parathyroid glands in mice (Chojnowski et al., 2014), but whose role in human cells has not yet been explored. The authors were able to demonstrate that HOXA3 regulates the differentiation of the inner coordination cells of the third pharyngeal pouch acting through the downstream Wnt signaling pathway by activating EPHB2 (Fu et al., 2024).

Together, these studies have helped define the pathophysiology of congenital athymia in humans. Additionally, several important advances have been made in the last few years in the field of biomaterials for the generation of thymic organoids. Some of these approaches have been nicely reviewed by Silva and colleagues (Silva et al., 2021). The most intriguing results in thymic organoids have been obtained using scaffolds mimicking the thymic ECM using only synthetic materials (e.g., porous tantulum-coated carbon matrix or polycaprolactone) (Palamaro et al., 2013; Poznansky et al., 2000), or a combination of synthetic and natural biomaterials (Hübscher et al., 2024, *Preprint*; Silva et al., 2021). However, the most successful approaches have been those using natural biomimetic materials, such as collagen scaffolds (Bortolomai et al., 2019), decellularized matrix-derived scaffolds (Asnaghi et al., 2021), or decellularized murine or rat thymic tissue (Campinoti et al., 2020; Fan et al., 2015; Hun et al., 2017; Ragazzini et al., 2023; Zeleniak et al., 2022), with the latest approaches using decellularized tissues providing exciting results when transplanted in vivo in mouse models. However, further studies on the best combination of cell types (directly isolated in vivo or differentiated from iPSCs), biomaterials, and culture conditions are still needed before the generation of transplantable thymic organoids could be envisaged as a future therapeutic approach (Fig. 2). Improvement in these models might also permit in the future to facilitate in vitro generation of both thymic stromal cells and T cells for therapeutic purposes.

## Conclusions and outlook

The advent of single-cell technologies allowed for the generation of multiple repositories and publicly available datasets ranging from fetal life to adulthood, both at the gene and protein level, as well as the epigenetic level. This provides a multiomics wealth of information that has advanced the current knowledge on thymus development, heterogeneity, and function in humans. The enormous amount of novel data obtained in the last few years has also generated new questions on various aspects of human thymus biology that can now be answered by taking advantage of the tools and datasets that have been created and that are publicly available to the scientific community. However, the identification of different cell types and subtypes poses the need to find common and shared definitions. For the hematopoietic compartment, such effort has been undertaken in the work by Domínguez Conde, Xu, Jarvis, Rainbow, Wells, et al., where a machine learning tool was developed for automated annotation of immune cells across human tissues, called CellTypist (Domínguez Conde et al., 2022). Employing this method in conjunction with thorough curation, the authors finely characterized immune cell types across various tissues, uncovering previously unnoticed tissue-specific characteristics and the clonal organization of T and B cells. Their comprehensive approach across multiple tissues establishes the groundwork for identifying consistently delineated immune cell types, utilizing a shared reference dataset, tissue-integrated expression analysis, and antigen-receptor sequencing. A similar approach and a universal definition for the stromal compartment is still lacking and will need to be the focus of future studies. While scRNA-seq studies have expanded our knowledge of gene expression patterns and led to the discovery of new TEC populations, there remains a pressing need to improve the identification of cell surface markers that are suitable for flow cytometry–based quantification, sorting, and ex vivo experimental studies of human TECs. To address this gap, Haunerdinger and colleagues (Haunerdinger et al., 2021) performed a screening of surface markers on thymic stromal cells and identified podoplanin as a general marker for human TEC, which, in combination with EpCAM, can distinguish these cells from other stromal cells, while the use of CD49f/ITGA6 and CD200 identify cTEC and mTEC, respectively (Haunerdinger et al., 2021). However, this study only covers a fraction of the newly characterized stromal cell subsets, for many of which specific surface markers are still lacking.

The introduction of spatial transcriptomics has allowed the field of thymus biology to make an enormous leap forward. However, an important downside of spatial transcriptomics data available in the literature on human thymus is that the resolution of the approach used in these reports was not at the single-cell level, making computational deconvolution necessary to identify cells included in each spot. This generates variability, depending on the computational approach selected, and also uncertainty in the predicted annotations, in particular when it concerns cells with high similarity, such as the thymocyte subsets. Novel approaches with increased resolution are becoming available (e.g., Slide-seq, Steroseq, and VisiumHD) (Chen et al., 2023; Oliveira et al., 2024, *Preprint*) and are expected to achieve higher degrees of reliability. Additionally, further studies using tissues isolated from older individuals and from diseased or dysfunctional thymic samples will be extremely helpful in validating the findings obtained in prenatal and early postnatal samples and will allow to gain critical insights into the processes of thymic development and involution. Together, this will lead to a better understanding of the causes of immunodeficiency and autoimmunity that characterize defects of T cell and thymic development and may ultimately result in novel therapeutic approaches for these conditions.

The quick advancement of both imaging and multiomics technologies provides the opportunity to elucidate some questions still outstanding in the field of thymus biology. Now that multiple subpopulations of cTECs and mTECs have been identified, understanding whether they retain specific locations in the thymus and what is their role in their neighborhood remains to be clarified. Additionally, characterizing the perivascular niche, the cortico–medullary junction, and the subcapsular area would potentially provide insights into TEC progenitors and their developmental trajectory from fetal to adult life. As for the thymocytes, divergent migration and maturation pathways between CD4 and CD8 cells have been described; understating the reasons behind this behavior and which signals are driving these differences would be important additions to further our understanding of immunity training. Other areas of research that would benefit from these novel high-resolution techniques include thymic involution during aging and thymic damage due to stress (e.g., chemotherapy, irradiation, and Graft versus Host Disease). Improved understanding of the cellular players involved in these processes could inform novel approaches to regenerate the thymus tissue.

Finally, it can be anticipated that the integration of in vitro models of T cell and TEC development together with datasets coming from an array of technological advancements may represent a first step toward the development of novel therapies for defects compromising thymus development and/or function, as well as thymic dysfunction associated with aging, autoimmunity, infections, and malignancies.
